# Semantic hyperspectral image synthesis for cross-modality knowledge transfer in surgical data science

**DOI:** 10.1007/s11548-025-03364-7

**Published:** 2025-04-24

**Authors:** Viet Tran Ba, Marco Hübner, Ahmad Bin Qasim, Maike Rees, Jan Sellner, Silvia Seidlitz, Evangelia Christodoulou, Berkin Özdemir, Alexander Studier-Fischer, Felix Nickel, Leonardo Ayala, Lena Maier-Hein

**Affiliations:** 1 Division of Intelligent Medical Systems, German Cancer Research Center (DKFZ), Heidelberg, Germany; 2 Helmholtz Information and Data Science School for Health, Karlsruhe/Heidelberg, Germany; 3 Faculty of Mathematics and Computer Science, Heidelberg University, Heidelberg, Germany; 4 National Center for Tumor Diseases (NCT), NCT Heidelberg, a Partnership Between DKFZ and University Hospital Heidelberg, Heidelberg, Germany; 5 Department of General, Visceral, and Transplantation Surgery, Heidelberg University, Heidelberg, Germany; 6 Medical Faculty, Heidelberg University, Heidelberg, Germany; 7 Department of Urology and Urosurgery, University Medical Center Mannheim, Medical Faculty of the University of Heidelberg, Mannheim, Germany; 8 Division of Intelligent Systems and Robotics in Urology (ISRU), German Cancer Research Center (DKFZ) Heidelberg, Heidelberg, Germany; 9 DKFZ Hector Cancer Institute at the University Medical Center Mannheim, Mannheim, Germany; 10 Department of General, Visceral, and Thoracic Surgery, University Medical Center, Hamburg-Eppendorf, Hamburg, Germany

**Keywords:** Knowledge transfer, Latent diffusion models, Semantic image synthesis, Hyperspectral imaging, Generative augmentation

## Abstract

**Purpose:**

Hyperspectral imaging (HSI) is a promising intraoperative imaging modality, with potential applications ranging from tissue classification and discrimination to perfusion monitoring and cancer detection. However, surgical HSI datasets are scarce, hindering the development of robust data-driven algorithms. The purpose of this work was to address this critical bottleneck with a novel approach to knowledge transfer across modalities.

**Methods:**

We propose the use of generative modeling to leverage imaging data across optical imaging modalities. The core of the method is a latent diffusion model (LDM) capable of converting a semantic segmentation mask obtained from any modality into a realistic hyperspectral image, such that geometry information can be learned across modalities. The value of the approach was assessed both qualitatively and quantitatively using surgical scene segmentation as a downstream task.

**Results:**

Our study with more than 13,000 hyperspectral images, partially annotated with a total of 37 tissue and object classes, suggests that LDMs are well-suited for the synthesis of realistic high-resolution hyperspectral images even when trained on few samples or applied to annotations from different modalities and geometric out-of-distribution annotations. Using our approach for generative augmentation yielded a performance boost of up to 35% in the Dice similarity coefficient for the task of semantic hyperspectral image segmentation.

**Conclusion:**

As our method is capable of augmenting HSI datasets in a manner agnostic to the modality of the leveraged data, it could serve as a blueprint for addressing the data bottleneck encountered for novel imaging modalities.

**Supplementary Information:**

The online version contains supplementary material available at 10.1007/s11548-025-03364-7.

## Introduction

Hyperspectral imaging (HSI) is an emerging, non-invasive surgical imaging modality that allows for the recovery of functional tissue information in real time, going beyond the limitations of conventional RGB (red, green, and blue)-based imaging. Recent evidence has shown that HSI not only outperforms RGB-based imaging in a number of clinical tasks such as disease classification and surgical scene understanding [1–3], but also enables entirely new applications, such as contrast agent-free intraoperative perfusion monitoring [4, 5] and cancer detection [6, 7].

**Fig. 1:**
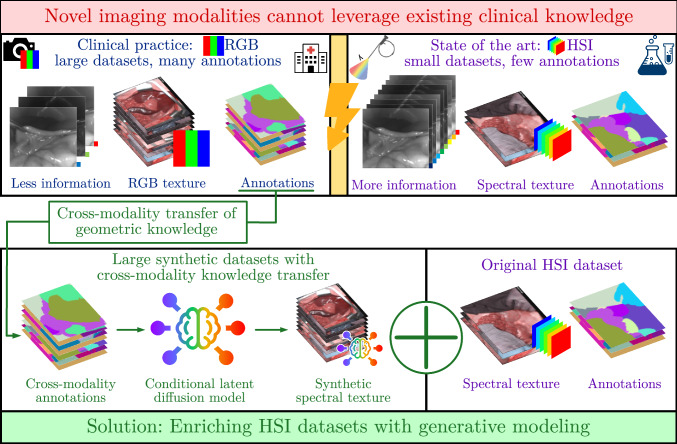
Our approach to leveraging imaging data across optical imaging modalities. The core idea is to train a modality-specific latent diffusion models (LDMs) for semantic hyperspectral image synthesis, capable of generating realistic labeled hyperspectral images from semantic annotations acquired from any modality

However, clinical translation of HSI and other novel imaging modalities is substantially hampered by the scarcity of high-quality annotated training data [8]. Especially as data from different imaging modalities cannot be directly integrated due to differences in spectral dimensions and appearance, data scarcity remains one of the core bottlenecks in the generalization of data-driven imaging algorithms. The use of data augmentation can, in principle, mitigate this issue [9]. However, in the context of segmentation, it suffers from a major conceptual drawback: To maintain accurate labels, both the segmentation masks and the images must be altered in a consistent manner. In the context of deformable augmentation, this can introduce problems when distorting texture according to distorted masks. This may be one major reason for why the surgical data science (SDS) community has so far only focused on rigid augmentation [10]. To overcome these limitations and inspired by recent breakthroughs in the field of generative augmentation outside the field of HSI [11, 12], we propose an alternative approach to enhancing existing HSI datasets, which uses generative modeling to convert a semantic segmentation mask obtained from any modality into a realistic labeled hyperspectral image (Fig. 1).

Our contributions can be summarized as follows:

1. *Cross-modality knowledge transfer*: We propose a novel concept for transferring knowledge across optical imaging modalities. Geometrical knowledge, originally obtained from conventional RGB images and encoded in segmentation masks, is transferred by conditioning an HSI synthesizer on the masks, as detailed in Fig. 1.
2. *Latent diffusion models (LDMs) in surgical HSI*: To instantiate the proposed concept, we pioneer the use of LDMs [13] for surgical HSI synthesis. Specifically, we show that LDMs can synthesize realistic surgical hyperspectral images of high resolution from semantic segmentation masks.
3. *Large-scale validation*: In a comprehensive validation study with a total of 13,489 HSI images annotated with an unprecedented number of 37 tissue and object classes, we show our method can be used as a generative augmentation approach capable of boosting the performance of semantic segmentation models applied to geometrical out-of-distribution (OoD) cases.

While a precise definition of OoD is non-trivial, we use the term OoD in this paper to refer to cases in which the tissue geometry is substantially altered by surgeons’ hands and instruments covering the surgical scene, by organs being isolated by cloth or by the image being blurred.

## Materials and methods

**Fig. 2:**
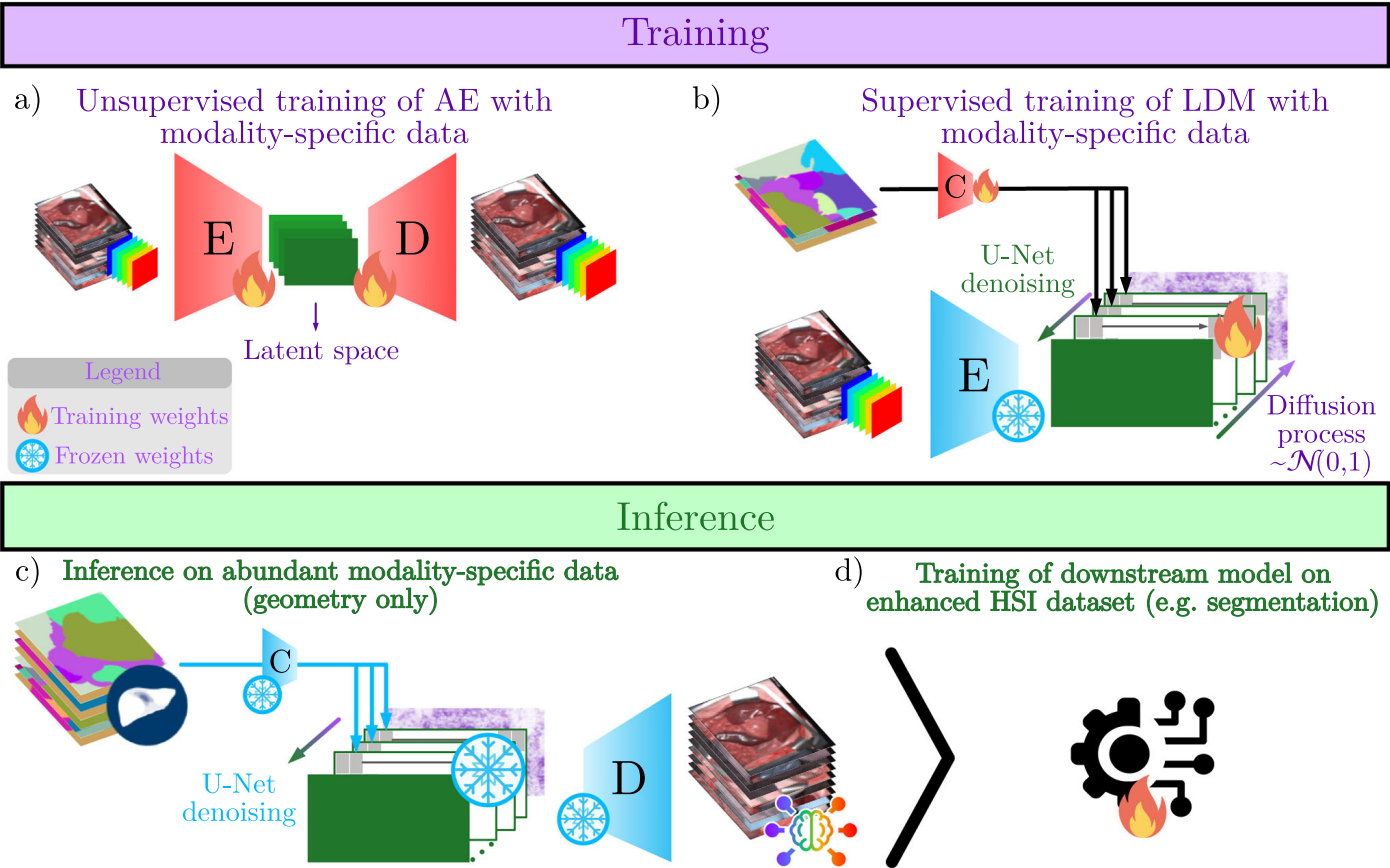
Concept overview. **a** To handle the high dimensionality of hyperspectral imaging (HSI) data, we first train an autoencoder (AE) to generate low-dimensional representations of HSI images. **b** The diffusion model (DM), conditioned on semantic segmentation masks, is then trained separately based on the frozen encoder of the AE. **c** Synthesis with segmentation masks obtained from any modality, and **d** applicable to any downstream task

Our approach to the semantic HSI synthesis is depicted in Fig. 2. In the upcoming sections, we present the LDM-based semantic image synthesis method (Sec. 2.1) and the experimental design (Sec. 2.2).

### Semantic image synthesis with latent diffusion models

To leverage annotated surgical scene data from different modalities (typically RGB), we propose applying a conditional LDM, consisting of an unconditional autoencoder (AE) and a conditional diffusion model (DM) [13] to semantic segmentation masks to generate HSI texture from otherwise unusable cross-modality annotations (Fig. 2). A key challenge in this context was the high dimensionality of the spectral data with 100 spectral bands next to the high spatial resolution. To address this challenge, we implemented the LDM with the following adaptations:

*AE Training* (Fig. 2a) In contrast to Rombach et al. [13], we modified the AE concept to compress not only the spatial dimensions but also the channel dimension, reducing it from 100 to 8. Our implementation also adapts the loss of the Kullback–Leibler (KL) regularized AE [13]: Empirical results during hyperparameter optimization showed that a lower regularization weight $\lambda _{\text {KL}}$ of the AE benefited the reconstruction quality. We removed the perceptual loss as it could not be directly applied to HSI images and omitted the adversarial loss as we did not observe any improvements in the reconstruction quality on our validation data.

*DM training* (Fig. 2b) DMs learn the data distribution by iteratively adding noise to transform the data to a simple distribution (typically $\sim \mathcal {N}(0, 1)$) and employ a model, commonly a U-Net, to learn the parameters required to revert the added noise [14]. As these algorithms do not depend on the dimensionality, we only made the following changes to the standard DM: We introduced padding to the non-quadratic latent space input to feed even resolutions on every downsampling layer in the internal DM U-Net architecture. To apply the semantic annotation as conditioning to the DM, we used the same approach as by Rombach et al. [13]: A conditioning encoder of a single convolutional layer and a simple spatial rescaling function was used to reduce the one-hot encoded segmentation maps to a tensor of shape 120 $\times$ 160 $\times$ 8, which is concatenated to the latent representation $z_t$ in every denoising step. The model implementation was done with the diffusers package [15], and its code repository can be found in: https://github.com/IMSY-DKFZ/hsi-diffusers.

*Inference* (Fig. 2c + d) HSI synthesis is achieved by using semantic segmentation masks (e.g., obtained from a different optical modality) as conditioning. We used the backward process of Song et al. [16] with 100 steps for faster sampling and classifier-free guidance (CfG) to balance the trade-off between the quality and diversity of the generated images under the specified conditioning [17]. We set the guidance scale to $\lambda _{\text {CFG}} = 2$ based on empirical analysis.

### Experimental setup

Our experiments addressed the following research questions (RQs):

*RQ1:* Can LDMs synthesize realistic, high-resolution surgical hyperspectral images with semantic segmentation masks as conditioning?
*RQ2:* Does the generative augmentation of HSI datasets benefit semantic segmentation models?

To this end, we used porcine datasets acquired under study IDs: 35$-$9185.81/G-161/18 and 35$-$9185.81/G-262/19. All datasets were acquired with a Tivita^®^ Tissue pushbroom camera (Diaspective Vision GmbH, Am Salzhaff, Germany), which spectrally resolves the $640 \times 480$ pixel images with 100 bands between 500 nm and 1000 nm, each with a width of 5 nm. Three different instances of the same camera model were used in the data acquisition process. All semantic annotations of the labeled datasets were either (i) created by two annotators with the annotation platform SuperAnnotate (SuperAnnotate, Sunnyvale, USA) [1], or (ii) by a team of medical experts with the Medical Imaging Interaction Toolkit platform [18]. For consistency, all annotations were revised by the same medical experts. The annotations contain up to 37 possible labels, including 19 tissue labels. For our experiments, we used the datasets described in Table 1 for method development, internal validation, and testing.

**Table 1:** Overview of development (upper) and untouched test sets (lower) used in this work

Name	# Img	# Subj	Purpose
DS-Train-Unlabeled	10,435	53	Training of the AE
DS-Val-Unlabeled	2472	10	Hyperparameter optimization of the AE
DS-Train-Semantic	157	6	Training of the DM
DS-Val-Semantic	50	3	Hyperparameter optimization of the DM
DS-Conditioning-ID	133	6	Generation of HSI images based on In-Distribution (ID) masks; includes a few masks with occlusion of tissue by surgeon’s hands
DS-Conditioning-OOD	24	7	Generation of HSI images based on OoD masks (cross-modality knowledge transfer); comprises masks with occlusion and isolation of tissue by surgeons’ hands and instruments
DS-Test-Semantic	93	5	In-Distribution (ID) testing on untouched test set
DS-Test-Occlusion	73	4	OoD testing on untouched test set; comprises images with occlusion of tissue by surgeons’ hands
DS-Test-Isolation	94	25	OoD testing on untouched test set; comprises images with isolation of tissue
DS-Test-Blur	115	1	OoD testing on untouched test set; comprises blurry images with occlusion and isolation of tissue by surgeons’ hands and instruments

For each dataset, the number of images, pigs, and the purpose of the dataset is specified

*Validation approach* Current research shows that chosen validation metrics in machine learning-based imaging often fail to reflect the clinical needs [19]. This holds especially true for the field of image synthesis, where validation guidelines are still lacking, and state-of-the-art (SOA) metrics [20, 21] are both dataset size- and domain-dependent and suffer from numerous pitfalls [22]. For this reason, we based our validation on three pillars: (1) qualitative assessment of visual realism, (2) quantitative assessment of realism through the analysis of spectral consistency, and (3) quantitative assessment using semantic segmentation as a downstream task.

*Addressing RQ1* We trained the AE on both DS-Train-Unlabeled and DS-Train-Semantic and fine-tuned the hyperparameters $\lambda _{\text {KL}}$ and the dimensions of the latent space on DS-Val-Unlabeled. Using the frozen AE, we then trained the DM on DS-Train-Semantic and used DS-Validation-Semantic to tune the dropout rate, amount of training steps, model size, and the guidance scale $\lambda _{\text {CFG}}$. To improve the model’s generalization from a small training set, we introduced an augmentation pipeline that included Rescaling, Rotation, Random-Crop, Random Flipping and Label Dropout [23]. If empty areas were created from an augmentation, we assigned that area to a new, unused label. For In-Distribution (ID) performance validation on an untouched test set, we synthesized HSI images using semantic masks from the test set DS-Test-Semantic. To simulate geometric knowledge transfer from another optical modality to HSI, we applied our HSI LDM to the masks in DS-Conditioning-ID and DS-Conditioning-OOD. Labels in DS-Conditioning-OOD not present in DS-Train-Semantic were reassigned to the background label “blue cloth”. To assess visual image quality, we reconstructed RGB images from HSI [24] and compared the generated with the real reconstructions.

*Addressing RQ2* We focused on semantic segmentation as our downstream task. To study the potential benefit of synthetic data, we used the state-of-the-art U-Net-based HSI segmentation architecture proposed by Seidlitz et al. [1]. We trained three models (i) on only DS-Train-Semantic (*Baseline*), (ii) only on synthetic data generated from the masks in DS-Conditioning-ID and DS-Conditioning-OOD (*Synthetic*), and (iii) on both (*Enhanced*). ID validation was performed with the DS-Test-Semantic dataset, and generalization capabilities were assessed with the OoD datasets DS-Test-Occlusion, DS-Test-Isolation, and DS-Test-Blur. Based on Maier-Hein et al. [25], we used the dice similarity coefficient (DSC) and the normalized surface distance (NSD) as validation metrics. The threshold for the NSD was set based on recommendations in Seidlitz et al. [1]. For a reliable statistical validation of the performance of the segmentation models on the test images, we decided to compute confidence intervals (CIs) on the performance metric differences between each model for each test dataset over conducting significance tests [26]. To calculate the CIs, we followed the recommendations in Jurdi et al. [27] and used bootstrapping. To account for the hierarchical structure of the data (multiple images per subject), we first calculated the mean metric values within each subject and then computed the average of these subject-specific mean values.

**Fig. 3:**
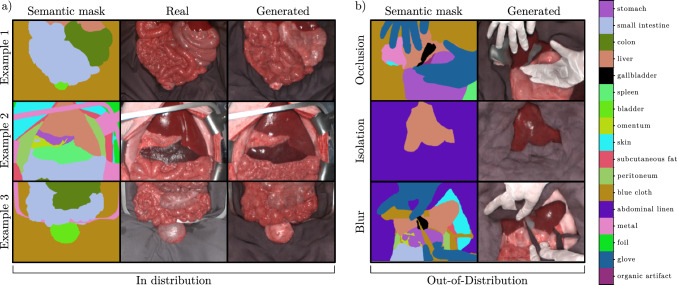
The synthetic hyperspectral images generated from semantic masks match their annotations well and visually and resemble real hyperspectral images. **a** Real images and masks taken from the ID test set and corresponding generated samples. **b** OoD masks with different settings, from both DS-Conditioning-ID and DS-Conditioning-OOD, and corresponding generated samples

## Results

Based on their RGB reconstructions with examples shown in Fig. 3, medical experts indicated that the generated images appear realistic. Specifically, the HSI images generated from semantic annotations possessed similar geometric features, similar lighting, shadow, and specular reflections, as well as similar organ color, organ-specific texture, and plausible organ boundaries compared to the real HSI images (Fig. 3a). However, we also observed differences to real images, particularly in background regions such as gloves, where finger shapes appeared implausible, and in organs with tubular structure such as the colon, where the colon walls were not well-connected. Additional differences were observed in finer details, including capillaries, which appeared to be fused together and inflamed, as well as major veins and muscles, which displayed incorrect texture. Similar qualitative observations can be made for the OoD dataset, with a lower degree of realism (Fig. 3b). Overall, the generated images appeared slightly blurred.

**Fig. 4:**
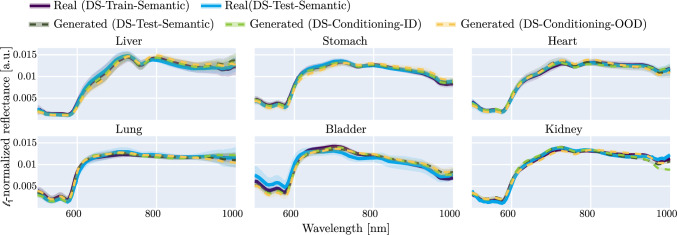
The median $\ell _1$-normalized spectra of generated and real images are in close agreement for each organ (here six exemplary organs). Our method shows very close agreement for most organs (liver, stomach), while under-represented organs (bladder, kidney) feature a lower spectral agreement. The normalized spectra of each dataset were hierarchically aggregated to obtain the mean and std across subjects

Figure 4 displays the aggregated, $\ell _1$-normalized spectra of the real and generated datasets for representative organs. For most classes, the spectra matched well according to their intensity values, local maxima, and minima. The best agreement was obtained for frequent labels such as liver and stomach, while the worst agreement was observed for rare labels such as kidney which only appears in 8 samples in the training set of 157 images.

**Fig. 5:**
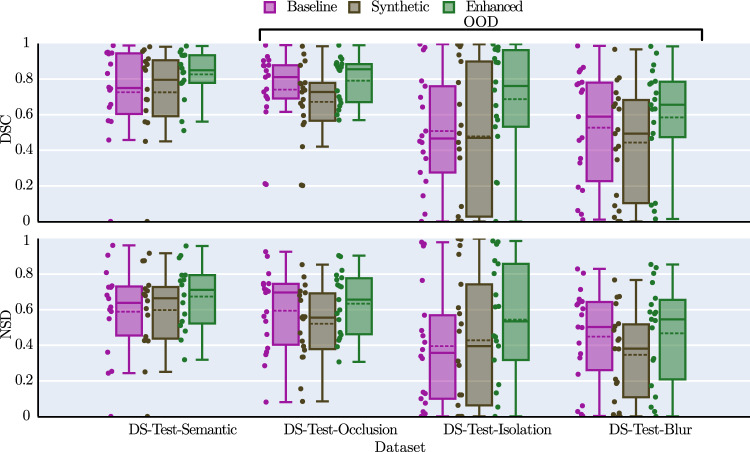
Generative augmentation improves performance in all settings according to the dice similarity coefficient (DSC) and normalized surface distance (NSD). Each point represents a mean estimate for DSC and NSD of a class label, respectively, which has been calculated from a per-subject hierarchical aggregation. We also included the downstream performance of the segmentation model *Synthetic* trained only on synthetic data, which is in some cases comparable with the *Baseline* model trained only on real data

Figure 5 shows the results of the downstream task. Generally, the *Enhanced* model has a higher mean and median performance than the *Baseline* model on each setting, with a mean relative performance boost ranging from 7% to 35% (in the DSC score) and from 4% to 37% (in the NSD score). Since the computed CIs of the performance metric differences between the models generally did not contain 0, the statistical validation also supports the observation that the *Enhanced* model outperformed the *Baseline* model. The *Synthetic* model trained with the same amount of only synthetic images as real images showed similar or slightly lower performance than the *Baseline* model.

## Discussion

To our knowledge, we are the first to propose an approach to leveraging geometry knowledge across imaging modalities in the field of SDS. In this context, we pioneered the application of LDMs for the synthesis of medical hyperspectral images. Our comprehensive study, comprising a total of 13,489 HSI images annotated with an unprecedented number of 37 tissue and object classes, suggests that LDMs are well-suited for semantic HSI synthesis even for OoD masks and are capable of leveraging geometry knowledge across modalities. Our study also suggests that our generative augmentation method enhances performance in both ID and OoD settings. These results are in line with findings from other medical domains and imaging modalities (RGB imaging) [28].

We are, to our knowledge, the first to apply LDMs in the field of surgical HSI. Works on HSI synthesis have so far been restricted to the field of remote sensing [29, 30]. The only works on LDMs in surgery that we are aware of were conducted with conventional RGB images rather than HSI and focused on image-to-image translation [31], text-to-image synthesis [32, 33], binary mask-to-video synthesis [34], and most recently semantic image synthesis [35]. The research gap on this topic is highlighted by the fact that a recent survey on DMs in medicine did not refer to any surgical work [36].

A limitation of our work could be seen in the fact that we did not leverage external datasets to show our method’s potential for cross-modality transfer. The reason is the general lack of publicly available data in SDS. However, we believe that semantic segmentation masks corresponding to different imaging modalities are highly similar, as geometric information of the tissue and therefore semantic annotations should be fairly agnostic to the applied image modality. Hence, we were able to simulate the setting of cross-modality transfer in a realistic manner.

Using preclinical porcine instead of clinical human data in our study could be considered another limitation. However, as hyperspectral imaging is not yet used in clinical routine, human data acquisition is extremely challenging and raises ethical concerns. In contrast, preclinical animal studies can be conducted in a controlled manner and can provide not only more but also standardized data. Since porcine models are commonly used for surgical training given the highly similar abdominal anatomy to humans [37], we adopted the common process of testing research hypotheses on porcine data.

We observed that LDMs do not model high-frequency details well and often generate slightly blurred images. In line with Isola et al. [38], we identified the AE as the cause for introducing this domain shift in the generated images. During the method development phase, we also observed replication of training data by the LDM despite applying a wide range of augmentations. However, as we work with a conditional LDM and provide different masks in training and testing, this was not a practical issue in our study.

In conclusion, we performed first steps toward leveraging LDMs for cross-modality knowledge transfer. Future work should not only concentrate on overcoming technical limitations such as AE quality and robust image quality assessment but also explore the transfer of other types of knowledge encoded in different modalities, such as knowledge related to depth, surface characteristics, and lighting conditions. Overall, our method could serve as a blueprint for addressing the training data bottleneck encountered for novel imaging modalities, thus enabling faster clinical translation to the benefit of both patients and clinical staff.

## Supplementary Information

Below is the link to the electronic supplementary material.

Supplementary file 1 (pdf 360 KB)
