# Supplementary material for: Semantic hyperspectral image synthesis for cross-modality knowledge transfer in surgical data science
Source: Int J Comput Assist Radiol Surg. 2025 Apr 24;20(6):1205–13. doi: 10.1007/s11548-025-03364-7 (PMC12167286; doi:10.1007/s11548-025-03364-7)
Supplement: Supplementary file 1 — (pdf 360 KB) [file 11548_2025_3364_MOESM1_ESM.pdf]

Supplementary Material
Semantic hyperspectral image synthesis for cross-modality knowledge transfer in surgical data science

International Journal of Computer Assisted Radiology and Surgery

Viet Tran Ba[1,3*†], Marco Hübner[1,3,4†], Ahmad Bin Qasim[1,2,3,4], Maike Rees[1,3], Jan Sellner[1,2,3,4], Silvia Seidlitz[1,2,3,4], Evangelia Christodoulou[1,4], Berkin Özdemir[5], Alexander Studier-Fischer[5,7,8,9], Felix Nickel[2,5,10], Leonardo Ayala[1,4†], Lena Maier-Hein[1,3,4,6†]

[1*]Division of Intelligent Medical Systems, German Cancer Research Center (DKFZ), Heidelberg, Germany.
[2]Helmholtz Information and Data Science School for Health, Karlsruhe/ Heidelberg, Germany.
[3*]Faculty of Mathematics and Computer Science, Heidelberg University, Heidelberg, Germany.
[4]National Center for Tumor Diseases (NCT), NCT Heidelberg, a partnership between DKFZ and University Hospital Heidelberg, Heidelberg, Germany.
[5]Department of General, Visceral, and Transplantation Surgery, Heidelberg University, Heidelberg, Germany.
[6]Medical Faculty, Heidelberg University, Heidelberg, Germany.
[7]Department of Urology and Urosurgery, University Medical Center Mannheim, Medical Faculty of the University of Heidelberg, Mannheim, Germany.
[8]Division of Intelligent Systems and Robotics in Urology (ISRU), German Cancer Research Center (DKFZ) Heidelberg, Heidelberg, Germany.
[9]DKFZ Hector Cancer Institute at the University Medical Center Mannheim, Mannheim, Germany.
[10]Department of General, Visceral, and Thoracic Surgery, University Medical Center, Hamburg-Eppendorf, Hamburg, Germany.

*Corresponding author(s). E-mail(s): viet.tranba@dkfz-heidelberg.de;
†Viet Tran Ba and Marco Hübner contributed equally to this work.
Leonardo Ayala and Lena Maier-Hein contributed equally to this work.

Table S1 Model and training hyperparameters.

Hyperparameter	Autoencoder	Diffusion model
GPUs	8 A100s (40 GB VRAM)	4 A100s (40 GB VRAM)
Batch size (per GPU)	1	2
VRAM usage (per GPU)	~ 29 GB	~ 28 GB
Training steps (per GPU)	$125,001$	$150,001$
Total learning rate	$8 * 10^{-5}$	$8 * 10^{-5}$
Optimizer	Adam $\beta_1 = 0.5, \beta_2 = 0.9$, Weight decay = 0.0	AdamW $\beta_1 = 0.9, \beta_2 = 0.999$, Weight decay = 0.01
Training time	30h	34h
Precision	Float32 precision	BFloat16 mixed precision
Model parameters	82M	446M
Dropout rate	0.0	0.2
$\lambda_{\text{Kullback-Leibler}}$	10^{-9}	-
Input space ($H \times W \times C$)	$640 \times 480 \times 100$	$120 \times 160 \times 8$
Latent space ($H \times W \times C$)	$120 \times 160 \times 8$	-
p_{uncond} (classifier-free guidance)	-	0.1
Conditioning	-	Annotation encoded to $120 \times 160 \times 8$ and concatenated in every denoising step
Zero padding	-	(4, 4, 0, 0) to obtain an input with spatial resolutions 128×160

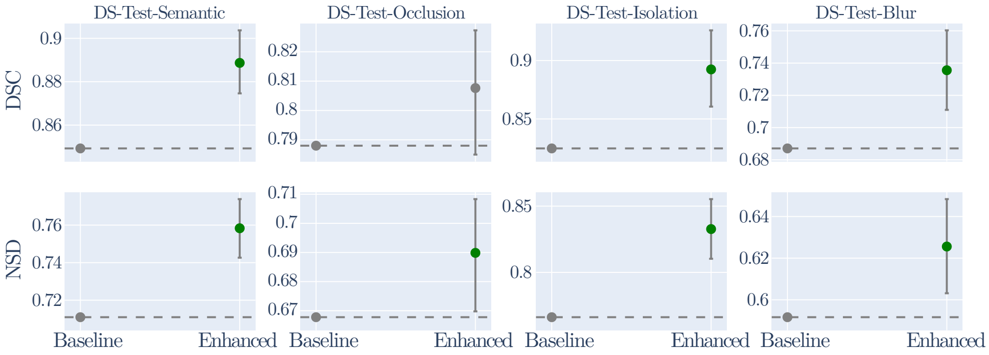

Fig. S1 Using generated hyperspectral images (generative augmentation) improves segmentation performance in both In-Distribution (ID) and Out-of-Distribution (OoD) settings. Comparison between the model without generative augmentation (*Baseline*) and with augmentation (*Enhanced*) was done by computing the score difference on each image in each test dataset. The error bars correspond to the confidence interval (CI)'s upper and lower bounds that were computed via bootstrapping [1], while the points have resulted from a per-subject hierarchical aggregation of the difference of metric values to the *Baseline* performance. For visualization purposes, the obtained CI bounds and means were shifted by the mean *Baseline* performance to show the absolute metric values.

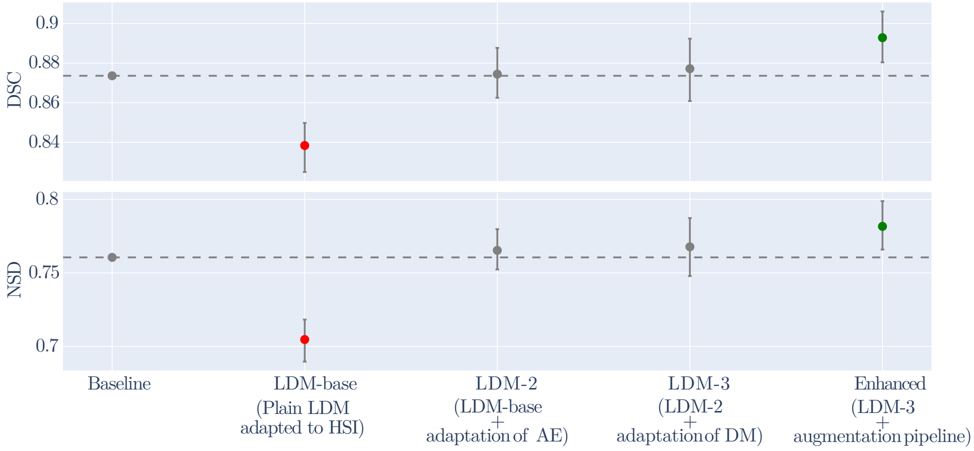

Fig. S2 Our model changes resulted in an incremental improvement in the downstream task performance. We validated our design proposals on the internal validation dataset by incrementally modifying the latent diffusion model (LDM) model. Downstream task models were trained on the DS-Train-Semantic dataset (*Baseline*), with additional data generated by: a plain latent diffusion model adaptation (*LDM-base*), a model incorporating autoencoder changes (*LDM-2*), diffusion model improvements (*LDM-3*), and the additional augmentation pipeline of Rescaling, Rotation, Random-Crop and Label Dropout [2](*Enhanced*). The error bars correspond to the confidence interval (CI)'s upper and lower bounds that were computed via bootstrapping [1], while the points have resulted from a per-subject hierarchical aggregation of the difference of metric values to the *Baseline* performance. For visualization purposes, the obtained confidence interval bounds and means were shifted by the mean *Baseline* performance to show the absolute metric values.

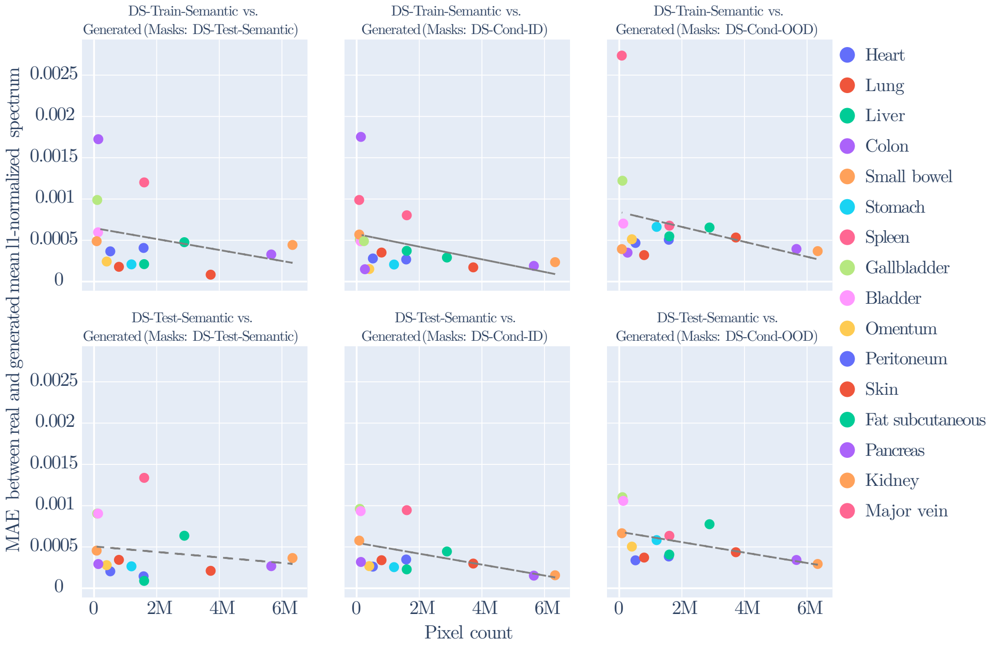

Fig. S3 The differences between the median ℓ_1-normalized spectra of generated and real images are very small, and there is a tendency for more common labels to have a smaller difference. Our method shows very close agreement for most organs, while underrepresented organs tend to feature a lower spectral agreement. To compute the differences between the real and generated datasets, the hierarchically aggregated spectra, partially presented in Fig. 4 of the main document, were compared.

References

[1] Jurdi, R.E., Varoquax, G., Colliot, O.: Confidence intervals for performance estimates in 3D medical image segmentation (2023)

[2] Islam, I., Puyol-Antón, E., Ruijsink, B., Reader, A.J., King, A.P.: Label dropout: Improved deep learning echocardiography segmentation using multiple datasets with domain shift and partial labelling. In: International Workshop on Advances in Simplifying Medical Ultrasound, pp. 112–121 (2024). Springer
